# Obstructive sleep apnea and stroke

**Published:** 2018-11-30

**Authors:** Shazia Jehan, Mahmoud Farag, Ferdinand Zizi, Seithikurippu R Pandi-Perumal, Alicia Chung, Anrew Truong, Girardin- Jean-Louis, Daniela Tello, Samy I McFarlane

**Affiliations:** 1 Department of Population Health, New York University School of Medicine, USA; 2 Department of Surgery, SUNY Downstate Medical Center, USA; 3 Somnogen Canada Inc., College Street, Canada; 4 Department of Medicine, SUNY Downstate Medical Center, USA

**Keywords:** OSA, CPAP, polysomnography, sleep, stroke, transient ischemic attacks, atrial fibrillation, cardiovascular disease, hypertension, dyslipidemia

## Abstract

Obstructive Sleep Apnea (OSA) is a common co-morbid condition in stroke patients. It represents a very important risk factor for stroke in addition to the other established ones such as hypertension, cardiovascular disease (CVD), hyperlipidemia, atrial fibrillation (AF), type 2 diabetes mellitus (T2DM), stress, smoking, and heavy drinking. Although in the United States the prevalence of OSA has somewhat decreased from the previous years, globally its prevalence remains constant, or in some cases, is on the rise. In this review we present the epidemiology for OSA in stroke populations and discuss the risk factors for stroke as well as the underlying pathogenetic mechanisms linking OSA, stroke and CVD. We also emphasize the more thorough evaluation and control of OSA in order to prevent the disabling side effects of a stroke, which not only compromises the physical and mental health of a person and increases the burden on families, but also adds a severe burden to national health economics. OSA should always be considered when assessing a patient with transient ischemic attacks (TIA). Work up and treatment for OSA will not only help prevent stroke with its devastating consequences, but will also help prevent CVD, and ameliorate co-morbid conditions such as diabetes and hypertension in these vulnerable populations.

## Introduction

OSA is a common finding in both stroke patients and in ischemic stroke patients; it is also an independent risk factor for all stroke patients.^1,2^ Given the prevalence of OSA, all stroke patients should be screened for OSA at the time of presentation of stroke using polysomnography.^3^ Knowing the etiology of a patient’s stroke offers a better opportunity to provide more effective treatment for stroke patients. Understanding the underlying cause of a stroke can help prevent recurrent strokes. One-third of strokes are the consequence of a patient’s previous history of stroke. OSA in stroke patients, if not treated, could lead to a recurrent stroke; therefore, addressing the management of OSA is a key to preventative health care in stroke patients.^4^ As OSA is an increasingly common finding in stroke patients, CPAP therapy has proven to have beneficial effectsin terms of improving neurological symptoms in stroke patients.^5^ Stroke patients with OSA have a worse prognosis overall, but treatment with CPAP can have a significantly better impact on overall cognitive and other physical disabilities suffered after stroke.^6^ Compliance with CPAP therapy improves overall neurological and physical health status in stroke patients with OSA.^7^

### Prevalence of OSA

OSA prevalence is increasing in developing countries due to an increased prevalence of obesity.^8^ The prevalence of OSA has been increasing over the past two decades.^9^ In order to address the risk of OSA and therefore the risk of stroke, the underlying issue of obesity must also be addressed (Table 1).

### Prevalence of stroke

The prevalence of stroke is strongly associated with OSA; particularly, the severity of OSA plays a significant role in the development of stroke.^10^ Stroke incidence increases with the severity of OSA.^11^ OSA is highly prevalent in stroke patients who are not treated with adequate CPAP treatment (Table 2).^12^

In a study conducted in Japan, researchers noticed a difference between patients with OSA and the control group. Patients with OSA showed silent cerebral infarction (SCI).^13^ Severe OSA, which occurs when the Apnea Hypopnea Index (AHI)≥15, shows higher prevalence of silent cerebrovascular lesions on MRI compared to less severe and moderate OSA. Therefore, polysomnography studies should be performed in all stroke patients regardless of the risk factors of OSA.^14^ Stroke mortality has decreased over the years in some of the countries which had the highest crude rate of mortality by stroke. This data is provided to the World Health Organization (WHO) by the Russian Federation, Ukraine, Belarus, Turkmenistan, and Kazakhstan. Because of advancement in healthcare and providing the better health to patients compared to the past, the mortality rate has significantly improved.^15^

### Risk factors for stroke

OSA is a risk factor for CVD.^16^ Additionally, OSA is a common risk factor of stroke in CVD patients.^17^ Severe OSA, which has an Apnea Hypopnea Index (AHI)≥30, is a major risk factor for stroke compared to less severe OSA, where the AHI is≥10.^18^ Along with OSA, high blood pressure, increased lipids, sedentary lifestyle, T2DM, and unhealthy eating habits are the major risk factors for stroke.^19^ Mohsenin^20^ studied patients with OSA who also reported having a stroke. The research mainly examined the involvement of the brainstem, and a polysomnography study showed more sleep disturbance in stroke patients having OSA compared to the control group. The severity of OSA has a bidirectional relationship with the severityof a patient’s initial symptoms of stroke and the clinical outcomes after stroke occurrence.^21^ Another study also emphasized this bidirectional relationship; OSA is more common in stroke patients regardless of neurological damage in the brain areas and lesions, and the severity of OSA plays an important role in stroke incidence.^22^ One prospective longitudinal study examining the elderly (age 70–100) shows that patients with severe OSA (AHI≥30) demonstrated higher incidence of stroke compared to patients without OSA.^23^ Stroke patients with OSA have a worse prognosis, experiencing a more prolonged hospitalization and spending more time in rehabilitation.^24^

OSA is an independent risk factor for stroke, even when controlling for other risk factors such as hypertension, diabetes mellitus, hyperlipidemia, and AF.^25^ The severity of the patient’s OSA apnea-hypopnea index (AHI≥30) plays an important role in the development of ischemic stroke, especially in older patients.^23^ OSA is associated with the neurological deficits in stroke patients; if OSA severity is≥25, then the severity of the stroke is aggravated.^26^ OSA is already present in many stroke patients prior to stroke occurring, regardless of any existing neurological deficits.^22^ OSA is a common finding in older male patients presenting with stroke and is associated with T2DM as well, contributing to a greater risk of death after stroke.^22^ There are several other risk factors of stroke other than OSA that have been reported. These major risk factors include, but are not limited to, T2DM, hypercholesterolemia, AF, hypertension, old age, and smoking.^27^ Another study also emphasizes that further risk factors for stroke are OSA, age, male sex, ethnicity, hypertension, andatrial fibrillation (AF).^28^ Along with OSA, Koo^19^ report that hypertension, resistant hypertension, T2DM, sedentary lifestyle, lack of exercise, smoking, and eating unhealthy food are the major and common risk factors for CVD and stroke. Dyslipidemia (85.9%) is also considered a major risk factor in stroke patients, especially the low HDL-C levels, followed by high blood pressure (66.0%), and T2DM (15.1%).^29^

**Figure 1:**
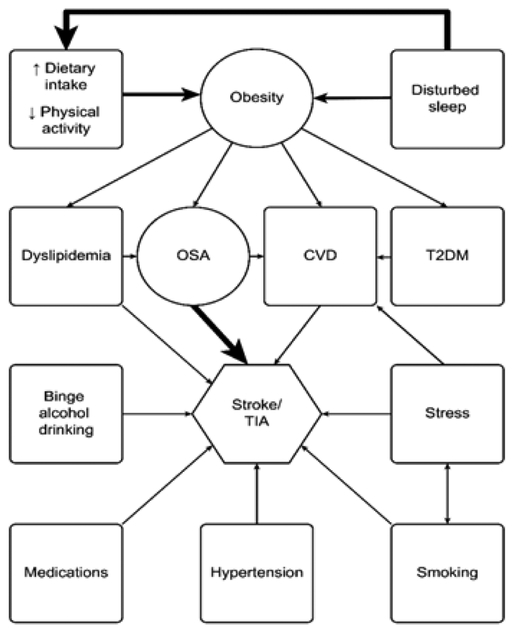
Risk factors for stroke

Cardiac arrhythmias are more common in OSA patients compared to those without OSA.^30^ AF was more common among OSA patients and had more stroke incidence compared to the control group.^31,32^ AF is one of the major risk factorsfor stroke, especially thromboembolic stroke. The underlying pathology of AF could worsen in the presence of OSA and other associated comorbidities such as high blood pressure and other cardiac myopathies.^33^ In OSA patients, cardioembolic (CE) strokes are more common when atrial fibrillation (AF) is present. When AF was treated with anticoagulation therapy, there was a lower incidence of stroke in these patients. The OSA severity and AF were directly proportional to the development of stroke (Figure 1).

### OSA and stroke pathophysiology

In OSA apneic/hypoxemic episodes initiate the process of inflammation and there is a cascade of inflammatory markers such as IL, 1, IL 6, TNF α, and interferon γ. These inflammatory markers damage the endothelial lining of the blood vessels, and there is an increased aggregation of platelets which lead to further oxidative stress and vascular endothelial damage. This repetitive oxidative stress and vascular damage in OSA patients can cause CVD and stroke.^34^ In OSA patients not only apneic/hypoxemic episodes cause oxidative stress and inflammatory damage to the blood vessels, but sympathetic system stimulation also releases catecholamines, and increased blood pressure leads to platelet aggregation and further damage to the vascular endothelium and progress to CVD and stroke.^35^ There is a dual effect of these apneic/hypoxic episodes; they not only stimulate the sympathetic system, but also depress the parasympathetic pathway. This inhibition r aids in the release of inflammatory markers, causing more oxygen desaturation, platelet aggregation, and endothelial damage. The sympathetic system activation is also responsible for hypertension, tachycardia, and myocardial wall dysfunction and damage.^36^

**Figure 2:**
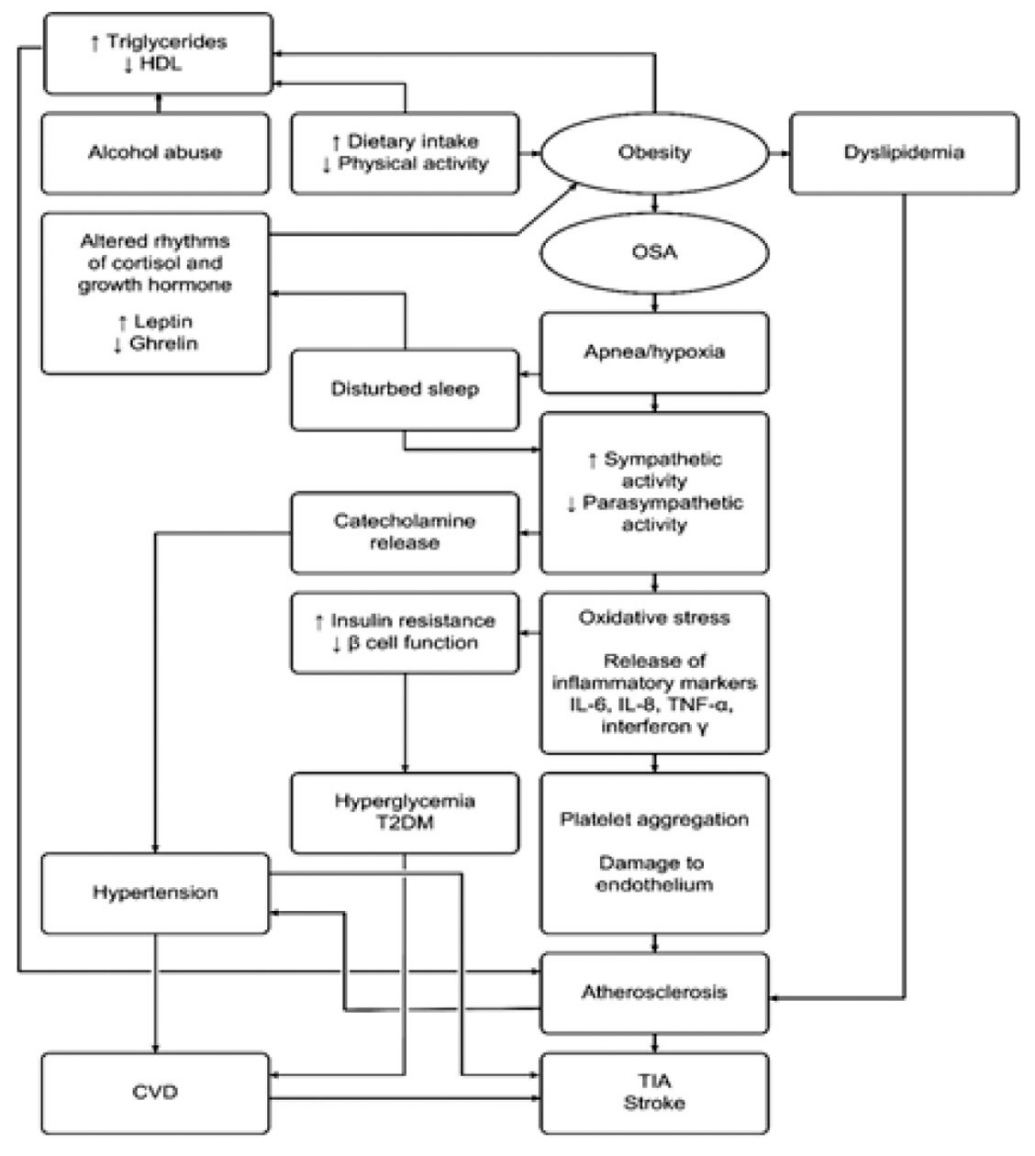
Pathogenesis of OSA, stroke and CVD

**Figure 3:**
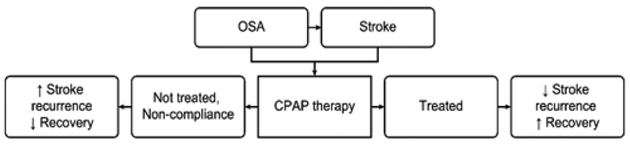
CPAP therapy and its outcomes in stroke and OSA patients

Because of these apneic/hypoxemic episodes in OSA patients, the activation of a sympathetic system and the beginning of the release of inflammatory markers predispose the patient to a stronger risk of CVD and stroke.^34^ As these apneic/hypoxemic episodes cause oxygen desaturation in OSA patients without any compensatory mechanism of anti-oxygenation, it leads to overproduction of reactive oxygen species, further oxygen desaturation and hypoxia causing ischemia in brain and leading to TIA and stroke.^37^ The severity of OSA is an independent risk factor for stroke and related mortality and morbidity.^25^ One study indicates that OSA could also be the causative factor of silent brain infarctions (SBI). Patients with moderate to severe OSA with SBI also showed increase in the inflammatory markers sCD40L and sP-selection, leading to CVD. When treating these patients with CPAP, there was a greater improvement in inflammatory markers sP-selection and CD40L.^38^ White matter is more affected by apneic/hypoxemic episodes in OSA patients; these episodes are a predisposing factor of stroke in these patients and their severity depends on the severity of OSA.^39^ Another study also shows that the pathogenesis of stroke is related to the severity of OSA; when the oxyhemoglobin saturation is less than 90% and AHI≥15 in stroke patients, then oxygen desaturation leads to white matter hyperintensities (WMH) in TIA and stroke patients (Figure 2 & Figure 3).^40^

### Treatment

OSA is a significant risk factor among stroke patients. Treatment of OSA with CPAP in stroke patients greatly improves the symptoms and effects of stroke. Diagnosis of OSA and treatment with CPAP have a greater impact on CVD and stroke; the diagnosis and treatment also improves the overall cognitive and physical disabilities caused by the patient’s stroke.^18,34^ Patients presenting with TIA and a minor stroke without showing any obvious signs and symptoms of OSA, such as elevated BMI, should also be screened for underlying OSA to prevent recurrence of stroke.^41^

A prospective observational study was carried out over 5 years on 166 patients who experienced stroke. These stroke patients were screened by polysomnographyfor OSA. The given treatment with CPAP in these stroke patients with severe and moderate sleep apnea demonstrated improvement in the overall health of these patients and better outcomes in terms of stroke mortality.^42^ The study shows there is a decreased incidence of new vascular events in stroke patients who are treated with CPAP compared to those in the non-treatment group.^43^ Treating stroke patients with OSA comorbidity with CPAP also decreases the risk of AF recurrence.^44^ The presence of OSA is a poor prognostic factor in stroke patients. Stroke patients presenting with OSA show more significant decline in cognitive functioning and overall functioning compared to patients without OSA. Additionally, they spend more time in rehabilitation centers.^45^ Treatment of stroke patients presenting with OSA using CPAP has greater benefits in cognitive and overall functioning.^46^ Although the treatment with CPAP greatly improves the depressive symptoms, neurological symptoms, and overall recovery in stroke patients with OSA, the compliance with CPAP therapy is a harder to maintain in stroke patients. Therefore, noncompliance could be a drawback in treatment of stroke patients with OSA.^47^ Treating stroke patients associated with OSA using CPAP is sometimes challenging; therefore an alternative approach to treating these patients should be considered.^48^ OSA is a major risk factor for stroke, high blood pressure, CVD, and other comorbidities. To overcome this growing problem, there is a need to control obesity, which is highly correlated with OSA.^49^ Smoking and alcohol consumption are also major risk factors in Western countries; to decrease stroke incidence, cessation of smoking and moderation of alcohol consumption should also be addressed.^50^

## Discussion

Obesity and OSA are both commonly rising health issues globally and should therefore be seriously addressed, counseled, and treated at each office visit with a primary care physician and other healthcare professionals.^49^ OSA is a very common finding in stroke patients, so every hospitalized patient with stroke and TIA should be screened for OSA.^51,52^ There is a strong correlation between inflammatory markers and the severity of OSA. In severe OSA, there is a more oxidative stress because of apneic/hypoxemic episodes, which cause the release of inflammatory markers such as IL6 (interleukin 6) and CRP (C-reactive protein). The release of inflammatory markers is directly proportional to the severity of AHI (Apnea/hypopnea index).^53^ An observational study showed that patients having OSA and CVD who were treated with CPAP had a better outcome compared to those not taking CPAP therapy.^54^ OSA is associated with the neurological deficits in stroke patients and also aggravates the severity of those deficits, which also depends on the severity of OSA.^26^ OSA is more common among minority groups, as shown in African Americans compared to Caucasian Americans, and has a greater association with CVD and stroke. The presence of OSA in stroke patients is a worse prognostic sign in terms of the cognitive and overall health of a patient. However, treatment with CPAP can have better overall health outcomes compared to the non-treatment group. Hence, lack of awareness and resources in this section of the population still prevent OSA treatment from being utilized effectively and efficiently. There is an increasing need for OSA awareness and treatment; the focus is necessary to prevent stroke and to gain better outcomes for stroke with OSA in the minority population, as well to improve the overall health and wellbeing of patients.^55^

Stroke prevalence in the United States has decreased in recent years. Stroke became the fourth leading cause of death, declining from its position as the third leading cause of death among the US population.^56^ However, among Asian countries, there are other risk factors which should be addressed and controlled to decrease the prevalence of stroke in these countries as well. Improved control of hypertension, T2DM, obesity, smoking cessation, regular exercise, and decreased salt intake can all help the overall health outcomes. There is a significant need for more stroke neurologists, diagnostic centers, and stroke treatment centers, which can decrease the incidence and prevalence of stroke in these countries as well.^57^ Controlling the risk factors of stroke can play an important role in reducing the incidence of stroke worldwide, as well as offering better control of hyperlipidemia and T2DM. Smoking reduction, avoidance of sedentary lifestyle, adopting a healthy lifestyle, eating healthy, maintaining a balanced diet, and exercising daily can reduce the risk of stroke.^58^

## Conclusion

Because OSA is one of the major risk factorsfor stroke and TIA, all stroke patients should be screened for OSA by using polysomnography studies. The prevalence of stroke is decreasing in the USA compared to past two decades because of efficient health care, as well as the increased availability of advanced laboratory investigations, skilled neurologists, and stroke rehabilitation centers. Additionally, in certain parts of Asia, its prevalence is decreasing, even in the countries which showed a higher prevalence according to the World Health Organization (WHO) in the past. Still, in most underdeveloped countries stroke morbidity and mortality are increasing due to the lack of health facilities, shortage of stroke specialists, limited availability of stroke centers, and less-advanced screening methods. Using CPAP to treat underlying OSA in stroke patients has more beneficial effects than in patients who are either not receiving the treatment or who are non-compliant with the treatment. Therefore, patients in less-developed countries are at a distinct disadvantage in terms of treatment of OSA and prevention of stroke. OSA is a highly emerging disease globally because of rising obesity; therefore, controlling obesity and more effectively managing OSA can offer a consistently better approach to treating stroke. Treating stroke patients with OSA is sometimes challenging, because of noncompliance with treatment, but there should be an alternative approach to treat these patients. CPAP therapy in stroke patients with OSA can be highly effective and beneficial. Adherence to CPAP therapy has a better prognosis and decreases the probability of recurrent stroke in OSA patients.

## Figures and Tables

**Table 1:** Prevalence of OSA worldwide

Age	Men	Women
30–70	24% – 26%	17% – 28%
30–49	10%	3%
50–70	17%	9%
30–60	24%	9%

**Table 2:** Prevalence of stroke world-wide

Design	Total n	% age	Age(years)	Men	Women
Prevalence of stroke and its risk factors in urban sri lanka: population-based study	2313	10.4 per 1000	≥18	−47.60%	−52.40%
Prevalence of stroke and coexistent conditions: disparities between indigenous and non indigenous Western Australians.	13 591	5%	25–84	33·7/1000	27·1/1000
Epidemiology of stroke in a rural community in Southeastern Nigeria	494	4.05 (n/1000), 4.41 men(n/1000), 3.75 women, (n/1000)	45–54	227	267
Epidemiology of stroke in a rural community in Southeastern Nigeria	414	12.08 (n/1000), 10.05 men (n/1000), 13.95 women (n/1000)	55–64	199	215
Epidemiology of stroke in a rural community in Southeastern Nigeria	329	6.08 (n/1000), 12.50 men, (n/1000), 0 women, (n/1000)	65–74	160	169
Stroke statistics in korea: part I. epidemiology and risk factors: a report from the korean stroke society and clinical research center for stroke	795,000	1.59%	≥30 years	411,000	384,000
Stroke prevalence in a poor neighbourhood of Sao Paulo, Brazil: applying a stroke symptom questionnaire.	4496	4·6% men, 6·5% women	35		

## Abbreviations:

OSA: obstructive sleep apnea
CVD: cardiovascular disease
TIA: transient ischemic attacks
AF: atrial fibrillation
SCI: silent cerebral infarction
AHI: apnea hypopnea index
